# Mortality and cost of therapy of carbapenem susceptible vs resistant Gram-negative infections among hospitalised patients in India: a multicentric surveillance (2022–2025)

**DOI:** 10.1016/j.lansea.2026.100829

**Published:** 2026-07-30

**Authors:** Nitin Bansal, Sonam Vijay, V. Ramasubramanian, Camilla Rodrigues, Prabhat Kumar, Bhawna Yadav, V.C. Ohri, Kamini Walia, ICMR AMR Network collaborators, Ambuj Yadav, Amit Gupta, Amit Kumar, Anju Susan Jacob, Aparna Chakraborty, Aparna Sonowal, Apurba Sankar Sastry, Arya Kumar, Ashish Bhalla, Ashok Kumar Pannu, Ashwani Gupta, Asim Kundu, Ayesha Sunavala, Babita Gupta, Balaji Veeraraghavan, Bashir Fomda, Bhanu Kumar, Bharti Taksande, Binila Chacko, Brijendra Kumar Rao, Chand Wattal, Chhavi Sawhney, Chiranjay Mukhopadhyay, D Himanshu Reddy, Daisy Phukan, Deepak Aggarwal, Deepak Gupta, Deepak Kumar, Deepthi Avvaru, Durga Shankar Meena, Gopal Krishana Bohra, Hari Prasad Sirigiri, Hitendrapal Solanki, Irom Loha, Ishrat Rashid, Jasper Rathinam, Jaswinder Kaur Oberoi, Kamal Uddin Zaidi, Kamran Farooque, Kapil Dev Soni, Kavitha Jayaram, Kiran Maribashetti, Mamta Kshetrimayum, Manisha Biswal, Meera John, Mitraa Shyam, Mousumi, Muralidhar Varma, N Sanjib Singh, Narayanan Parameswaran, Nasima Ahmed, Navneet Sharma, Neelam Taneja, Nishad Plakkal, Nusrat Shafiq, Padmaja Durga, Pankaj Arora, Parijat Das, Pradeep Saxena, Pralay Ghosh, Pranjal Kumar Dutta, Prashant Gupta, Purva Mathur, Raja Ray, Reema Nath, RehanUl Haq, Richa Aggarwal, Ritin Mohindra, Roohi Mohiuddin, Rupamdeep Kaur, Sadia Khan, Sadik Mohammed, Sagar Khadanga, Sai Shree, Saidulu Ganta, Samir Yelwatkar, Sanjeev Singh, Sayli Gore, Sheetal Varma, Subhalakshmi Das, Subodh Kumar, Sudipta Mukherjee, Sukanya Vimala, Sushma Sagar, Syed Mudasir Qadri, Tadepalli Karuna, T Jeeten Kumar Singh, Vandana Kalwaje Eshwara, Vanita Jain, Venkateswaran Ramanathan, Vibhor Tak, Vijay Sharma, Vineeta Dhyani, Vijayshree Deotale, Vivek Handa, Vivek Trikha, Wazid Ali, Yamunadevi V. Ramanathan, Yogiraj Roy

**Affiliations:** a Division of Descriptive Research, Indian Council of Medical Research, Ansari Nagar, New Delhi, 110029, India; b Department of Infectious Diseases, Apollo Hospitals, Greams Rd, Chennai, 600006, Tamil Nadu, India; c Department of Microbiology, PD Hinduja Hospital, Mahim, Mumbai, 400016, Maharashtra, India; d Division of Development Research, Indian Council of Medical Research, Ansari Nagar, New Delhi, 110029, India

**Keywords:** Antimicrobial surveillance, Carbapenem resistance, Gram-negative pathogens, Tertiary-care, Clinical outcome, Economic burden

## Abstract

**Background:**

India bears one of the highest burdens of Gram-negative bacterial infections, yet their burden and associated mortality remain poorly characterised. We aimed to determine and compare the mortality and cost of therapy for susceptible and resistant Gram-negative bacterial infections from a multicentric hospital-based surveillance in India.

**Methods:**

This multicentre observational study analysed ICMR antimicrobial resistance surveillance network data from April 2022 to April 2025 across 20 tertiary care hospitals in India. We enrolled hospitalised patients with microbiologically confirmed gram negative infections caused by *Escherichia coli*, *Klebsiella pneumoniae*, *Acinetobacter baumannii*, and *Pseudomonas aeruginosa*. Mortality among patients with carbapenem resistant infections was compared with phenotypically susceptible infections of the same species. Secondary outcomes included length of stay, cost of therapy and treatment patterns with ceftazidime–avibactam or polymyxin based therapy.

**Findings:**

Among 159,336 hospitalised patients (1,759,597 patient-days), 27,283 (17.1%) were microbiologically confirmed and 26,213 (16.4%) patients had Gram-negative bacterial infections. The mean age of the study population was 51.2 years (SD 18.3), and 67.8% of them were males and 32.2% were females. Patients with carbapenem-resistant (CR) infections were associated with a higher risk of mortality than susceptible infections across all major pathogens studied: *E. coli* [relative risk (RR) 1.41, 95% CI 1.13–1.70; population attributable fraction (PAF) for mortality 15.8%], *K. pneumoniae* [RR 1.33, 95% CI 1.18–1.51; PAF 17.5%], *A. baumannii* [RR 1.16, 95% CI 1.02–1.34; PAF 11.1%], and *P. aeruginosa* [RR 1.43, 95% CI 1.15–1.92; PAF 18.7%]. Bloodstream infections due to carbapenem resistant non-fermenters had the highest mortality (46.4–50.8%). CR *E. coli* and *K. pneumoniae* were predominantly treated with ceftazidime–avibactam over polymyxin B. Antibiotic treatment costs were 1.1–2 folds higher for resistant infections.

**Interpretation:**

Out findings suggest that CR Gram-negative infections are associated with high mortality and cost of therapy in tertiary care hospitals in India. These findings underscore the urgent need to strengthen surveillance, infection prevention and control and to improve access to effective therapies through strong antimicrobial stewardship initiatives.

**Funding:**

Task force project of the Indian Council of Medical Research.

**Research in context**

**Evidence before this study**

We searched PubMed database without language restrictions, using the terms *“Gram-negative pathogens”*, *“burden”*, *“carbapenem-resistant” and hospitalised patients* to identify studies assessing the burden of carbapenem-resistant (CR) Gram-negative infections. We included studies published between April 1, 2026, and June 1, 2026. The available evidence from 30 studies is limited and heterogeneous, largely restricted to single-centre studies, specific populations (e.g., neonatal sepsis), or focused on microbiological or molecular epidemiology. Existing systematic reviews and multi-country studies have identified CR Gram-negative bloodstream infections as a major contributor to mortality, with a disproportionately high burden in South Asia, including India. A study from Vietnam reported CR Gram-negative bacterial infections using a standardised, online, cross-sectional questionnaire approach. Most published studies from India have reported isolate-level microbiological data; we did not identify any multicenter studies from India that assessed burden, treatment modalities and mortality associated with CR Gram-negative infections.

**Added value of this study**

This multicentre observational study analysed ICMR antimicrobial resistance surveillance network data from April 2022 to April 2025 across 20 tertiary care hospitals in India. We aimed to determine and compare the mortality and cost of therapy for susceptible and resistant Gram-negative bacterial infections among hospitalised patients. A total of 159,336 hospitalised patients (1,759,597 patient-days) were included, 27,283 (17.1%) were microbiologically confirmed and 26,213 (16.4%) patients had Gram-negative bacterial infections. Patients with CRt infections were associated with a higher risk of mortality, prolonged hospitalisation, and increased treatment costs than susceptible infections across all major pathogens (*Escherichia coli*, *Klebsiella pneumoniae*, *Acinetobacter baumannii*, and *Pseudomonas aeruginosa)* studied, particularly for non-fermenting Gram-negative pathogens.

**Implications of all the available evidence**

The available evidence indicates that CR Gram-negative infections are associated with excess mortality, prolonged hospitalisation, and increased treatment costs in India. These findings underscore the urgent need to strengthen integrated AMR surveillance that links microbiological data with clinical outcomes. There is also a critical need to expand infection prevention and control practices and implement targeted antimicrobial stewardship programmes to promote the rational use of antibiotics. In parallel, improving access to timely diagnostics and effective, affordable novel antimicrobials will be essential to improve outcomes and reduce the economic burden of these infections for patients.

## Introduction

Antimicrobial resistance (AMR) has emerged as one of the major global health challenges, contributing to an estimated 9% of deaths worldwide.^1^^,^^2^ Recent estimates indicate that bacterial AMR was associated with 4.95 million deaths in 2019 and 4.71 million deaths in 2021, with over 1.1 million deaths attributable to AMR.^2^^,^^3^ Among the Gram-negative pathogens, carbapenem resistance is of particular concern due to limited treatment options and poor outcomes. Recent global analyses indicate that South Asia, including India, bears one of the highest burdens of carbapenem-resistant (CR) bloodstream infections and related mortality.^4^

The evidence on burden of drug-resistant infections in low-income and middle-income countries (LMICs) like India, remain limited due to fragmented surveillance, incomplete laboratory and clinical data, and absence of standardised methods for estimating mortality and economic impact. The 2019 AMR burden study identified six pathogens, *E. coli*, *Staphylococcus aureus*, *K. pneumoniae*, *Streptococcus pneumoniae*, *A. baumannii*, and *P. aeruginosa,* being collectively responsible for over 2.5 million deaths.^2^ Several of these pathogens are designated as priority pathogens by the WHO because of their association with healthcare-associated infections and their escalating multidrug resistance. CR *E. coli*, CR *Acinetobacter baumannii* (CRAB), and CR *P. aeruginosa* (CRPA) are associated with prolonged hospitalisation, limited treatment options, and increased morbidity and mortality.^5^

In India, data from the Indian Council of Medical Research (ICMR) – antimicrobial resistance surveillance network have documented rising resistance to carbapenems and other antibiotics among key Gram-negative pathogens including *K. pneumoniae*, *A. baumannii*, and *P. aeruginosa* which are commonly implicated in hospital-acquired infections in the tertiary care hospitals.^6^^,^^7^ However, most available evidence in India has been limited to isolate-level microbiological surveillance, antimicrobial susceptibility patterns, or molecular characterisation, with limited availability of multicentre patient-level data on clinical outcomes and cost of illness.^8^

Understanding the patient-level burden associated with carbapenem resistance is essential to inform antimicrobial stewardship initiatives, infection prevention strategies, and healthcare policy. Therefore, this multicentre study utilised data from a hospital-based surveillance network in India across tertiary care hospitals. We aimed to generate surveillance-level estimates of mortality, length of hospitalisation, and treatment costs associated with CR infections compared with CS infections of the same species.

## Methods

This multicentre hospital-based observational study was conducted from April 2022 to April 2025 across 20 tertiary care hospitals in India. This study was undertaken within the multicentre AMR surveillance network. This study was conducted in accordance with the Strengthening the Reporting of Observational Studies in Epidemiology (STROBE) reporting guidelines. As the analysed data were collected within the framework of a broader network with multiple objectives, detailed patient-level severity indicators (e.g., severity scores, organ support measures, and longitudinal clinical variables) were not uniformly captured across participating centres.

Participating hospitals were selected to ensure broad geographical representation across various regions of India (Table S1). Each participating hospital was required to designate a minimum of 150 inpatient beds for surveillance activities, including at least 50 intensive care unit (ICU) beds and 100 ward beds. All patients, irrespective of age or gender admitted to these designated beds were prospectively followed until their final clinical outcome (discharge or death). These beds were selected to facilitate uniform prospective surveillance across all the participating hospitals while ensuring operational feasibility. Continuous follow-up of all the hospitalised patients was not feasible because of resource and personnel constraints. Relevant microbiological, clinical and outcome data were entered into a centralised data management portal to ensure harmonised data collection across all centres.

Microbiology laboratories at participating hospitals followed standardised operating procedures (SOP) developed by the Indian Council of Medical Research with antimicrobial susceptibility testing being performed following either Clinical and Laboratory Standards Institute (CLSI) 29th edition or European Committee on Antimicrobial Susceptibility Testing guidelines, as applicable.^9^

Patients considered eligible were those with microbiologically confirmed infections caused by pathogens such as *A. baumannii, K. pneumoniae, P. aeruginosa,* and *E. coli.* Each resistant infection (“R” group) was compared with infections caused by phenotypically susceptible isolates of the same species (“S” group). CR isolates were defined phenotypically as isolates reported as resistant (R) to at least one tested carbapenem agent according to CLSI breakpoints.^10^ Intermediate (I) isolates were not classified as resistant or non-susceptible and were therefore not included in the CR group. CS isolates were defined as isolates susceptible to all tested carbapenems. Microbiologically confirmed infections were classified as community-acquired if the culture was positive within 48 h of hospitalisation and there was no history of hospitalisation within the preceding 90 days. Infections not meeting these criteria were classified as healthcare-associated infections.

The classification of infection versus colonisation was primarily based on the clinical judgement of the treating physician in conjunction with microbiological findings at the bedside. However, broad syndromic definitions were included within the AMR–AMS network study protocol to support clinicians in differentiating true infection from colonisation and to promote consistency across sites. The relevant definitions are provided in Supplementary File.

A centralised data management system with a secure, password-protected online portal was developed specifically for this project. Each participating hospital had unique access credentials and was responsible for entering patient-level data using variables pre-defined in the study protocol. Personnel responsible for data entry underwent structured trainings organised by ICMR to ensure accuracy in data entry. Data were collected prospectively and entered in real time. Data confidentiality was maintained by encrypting all personally identifiable information. Periodic data quality checks, validation audits, and feedback loops were conducted to check for inconsistencies and ensure completeness.

The following patient variables were recorded: demographic data (age, gender), underlying medical conditions including co-morbidities and primary diagnosis, microbiological data, antibiotics consumption, clinical outcomes [length of hospital stay (calculated from date of admission in the hospital to date of outcome i.e., discharge or death), in-hospital mortality]. The population attributable fraction (PAF) for mortality was also computed. The cost of treatment referred in the manuscript includes only the cost of antibiotics available under the JanAushadhi Scheme (Government of India) (Table S2).

The study also evaluated the therapeutic regimens administered to patients with carbapenem resistant infections. The patients were either treated with polymyxin-based therapy which included the use of colistin or polymyxin B, either as monotherapy or in combination with other antimicrobial agents or ceftazidime–avibactam–based therapy comprising of ceftazidime–avibactam alone or in combination with aztreonam. Treatment duration and clinical outcomes were captured for all the antimicrobial regimens used. Comparative analyses were conducted between polymyxin-based and ceftazidime–avibactam–based therapies for infections with carbapenem resistant organisms.

### Statistical analysis

Comparison between the resistant and susceptible infection groups was undertaken to assess the impact of drug resistance on the clinical outcomes and healthcare costs. Continuous variables were summarised as mean and standard deviation (SD) as appropriate based on data distribution and were compared using t-tests or chi-square tests. Differences in proportions with 95% CI were calculated to assess differences in comorbidity prevalence between groups. A two-sided *p* value <0.05 was considered statistically significant. Relative risk (RR) with 95% CIs was calculated to estimate the association between carbapenem resistance and mortality. Confidence intervals and p-values for mortality analyses were adjusted for clustering by study site using modified Poisson regression, in which the binary outcome (death vs. survival) was modelled with a Poisson log-link function. The regression coefficient β for carbapenem resistance represents the log risk ratio; the exponentiated coefficient exp(β) is reported as the RR with its 95% confidence interval. All analyses were performed using Stata/MP version 18.0.

The population attributable fraction (PAF) for mortality was calculated using Levin's formula: PAF = Pe × (RR − 1)/[Pe × (RR − 1) + 1], where *Pe* represents the proportion of patients with resistant infections among all patients with a particular infection, and RR denotes the relative risk (unadjusted RR was used for PAF estimation). The 95% confidence interval for PAF was calculated using Delta method, where number of carbapenem resistance within each pathogen category used as the sample size. Variance of PAF (V[PÂF]) is estimated from the variances of the Pe estimator (V[Pe]) and β (beta estimator), as well as the RR and Pe.^11^ The detailed formula used for the calculation is provided in Supplementary File.

### Ethics statement

The study was approved by Ethics Committees of all the participating hospitals; approval numbers are provided in Supplementary File. The requirement for consent was waived as the data were analysed while maintaining full confidentiality after removing all the patient identifiers. The study was conducted in accordance with the Declaration of Helsinki.

### Role of the funding source

The funding agency, ICMR, designed and implemented the study through its network of 20 participating hospitals.

## Results

**Fig. 1:**
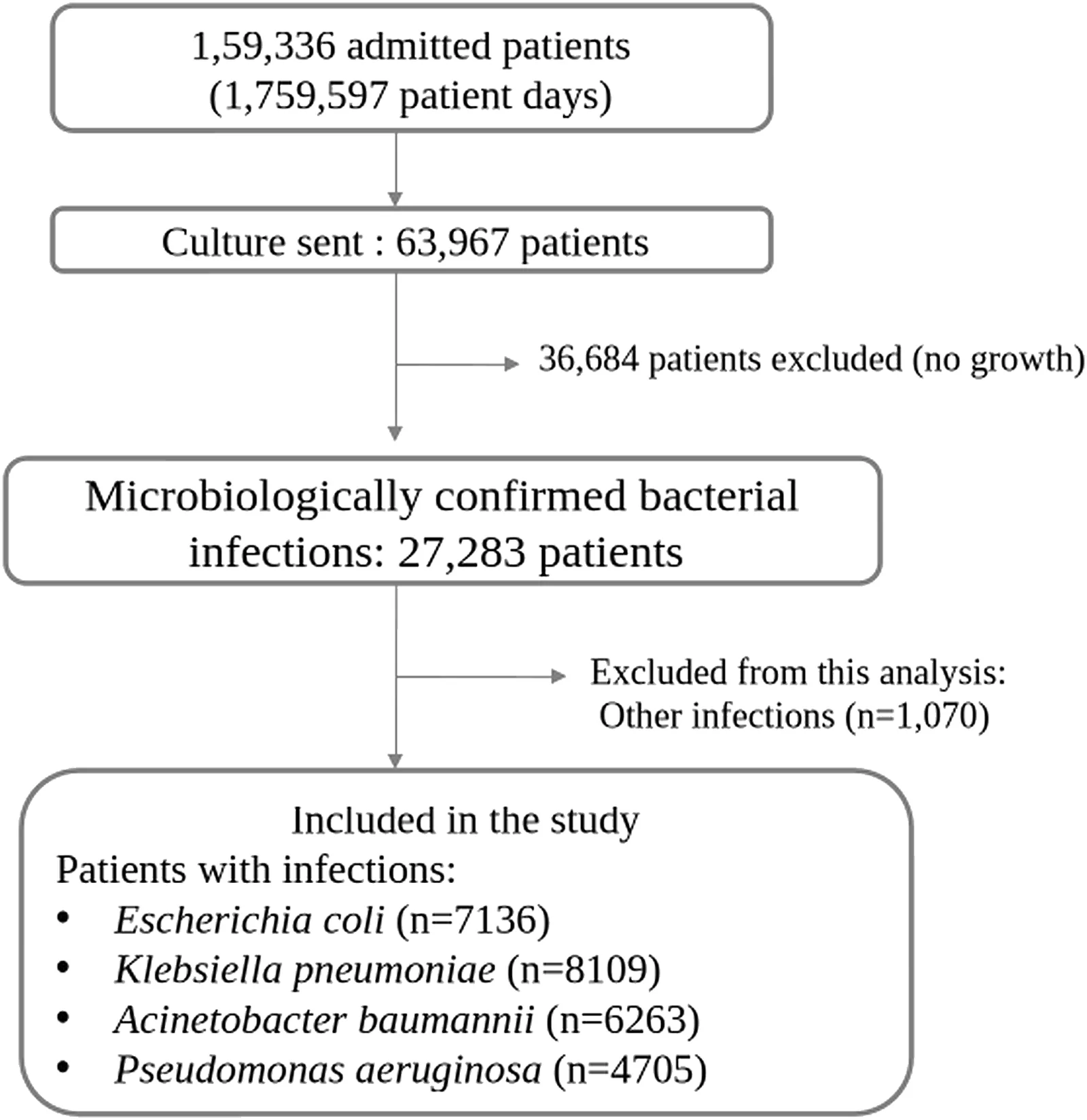
Study flow diagram of patients.

Between April 2022 and 2025, 159,336 patients corresponding to a total of 1,759,597 patient-days, were enrolled across 20 tertiary care hospitals participating in the network. Of these, 26,213 (16.4%) patients had Gram-negative bacterial infections. The mean age of the study population was 51.2 years (SD 18.3), and 67.8% of them were males and 32.2% were females. Among patients with Gram-negative infections, 16,025 (61.1%) had CR infections. Diabetes mellitus and the presence of a luminal device were more common among patients with CR infections, whereas active malignancy was more frequently observed in the CS group. *K. pneumoniae* was the predominant pathogen among patients with CR infections, while *E. coli* was the most frequently isolated pathogen in the CS group. The median length of hospital stay was comparable between the two groups. However, in-hospital mortality was higher among patients with CR infections than those with CS infections (Table 1). Patients with a culture-positive clinical specimen growing *E. coli*, *K. pneumoniae*, *P. aeruginosa*, or *A. baumannii* at any time during their hospital stay were included in the subsequent analysis. Patients with infections other than these four organisms were excluded. Duplicated records and patients with polymicrobial infections were excluded from the analysis. Fig. 1 provides details on number of included samples across infections.

**Table 1:** Characteristics, comorbidities and clinical outcomes among study population.

	All patients	Carbapenem resistance n (%)	Carbapenem sensitive n (%)	P value
	N (%)			
Age (in years) Mean (SD)	51.2 (18.3)	50.5 (18.7)	52.2 (19.1)	<0.0001
Gender				
Male	17,765 (67.8)	11,025 (68.8)	6740 (66.2)	
Female	8448 (32.2)	5000 (31.2)	3448 (33.8)	<0.0001
Diabetes mellitus	7655 (29.2)	4519 (28.2)	3136 (30.8)	<0.0001
Presence of luminal device	4194 (16.0)	3106 (19.4)	1088 (10.7)	<0.0001
Chronic kidney diseases (CKD)	3893 (14.9)	2632 (16.4)	1261 (12.4)	<0.0001
Active malignancy	3984 (15.2)	1730 (10.8)	2254 (22.1)	<0.0001
Recent surgery (within 90 days)	2995 (11.4)	2108 (13.2)	887 (8.7)	<0.0001
Pathogen distribution				
*E. coli*	7136 (27.2)	3240 (20.2)	3896 (38.2)	<0.0001
*K. pneumoniae*	8109 (30.9)	5305 (33.1)	2804 (27.5)	<0.0001
*A. baumannii*	6263 (23.9)	4977 (31.0)	1286 (12.6)	<0.0001
*P. aeruginosa*	4705 (17.9)	2503 (15.6)	2202 (21.6)	<0.0001
Hospital associated infection				
*E. coli*	6068 (25.5)	2898 (18.0)	3170 (31.1)	<0.0001
*K. pneumoniae*	7432 (31.2)	4945 (30.8)	2487 (24.4)	<0.0001
*A. baumannii*	5958 (25.1)	4730 (29.5)	1228 (12.0)	<0.0001
*P. aeruginosa*	4326 (18.2)	2355 (14.6)	1971 (19.3)	<0.0001
Length of hospital stay Mean (SD)	23.5 (22.3)	24.3 (22.4)	23.0 (22.4)	<0.0001
Death	7254 (27.7)	5057 (31.6)	2197 (21.6)	<0.0001

Data are presented as mean ± standard deviation (SD) for continuous variables and as n (%) for categorical variables. Continuous variables (Age, length of stay) were compared using the independent-samples *t* test, while categorical variables (Gender, co-morbidities), Death were compared using the Pearson's Chi–Square Test of Independence. All tests were two-sided, and a *P* value <0.05 was considered statistically significant.

Among the 7136 total *E. coli* infections, 3240 were CR *E.coli*, while 3896 were CS *E.coli*, 6068 infections (85%) were classified as healthcare-associated. Among the 8109 *K. pneumoniae* infections, CR (CRKP) isolates were identified in 5305 and CS (CSKP) in 2804 patients and 7432 infections (91.6%) were classified as healthcare-associated infections. Among 6263 patients with *Acinteobacter baumannii* infections, 4977 were CRAB, 1286 were CS *Acinetobacter baumannii* (CSAB) and 5958 infections (95.1%) were classified as healthcare-associated infections. Among 4705 patients infected with *P. aeruginosa*, 2503 patients were CR *P. aeruginosa* (CRPA), 2202 had CS *P. aeruginosa* (CSPA) and 4326 infections (91.9%) were classified as healthcare-associated infections (Table 1). Among blood stream associated infections across all pathogens, again majority (more than 85%) were classified as healthcare-associated infections.

The mean age and gender distribution across all pathogens are summarised in the respective tables and they are similar across both CR and CS groups (Table 2). The presence of luminal device and recent surgery (within 90 days) were higher across resistant groups of all pathogens, suggesting a healthcare-associated risk. CKD and Diabetes mellitus was higher among CRAB *and* CKD was higher among *P. aeruginosa* (Table 2).

**Table 2:** Pathogen wise demographic characteristics and comorbidities among patients infected with gram-negative infections.

	*E. coli*		*K. pneumoniae*		*A. baumannii*		*P. aeruginosa*	
	CR n (%)	CS n (%)	CR n (%)	CS n (%)	CR n (%)	CS n (%)	CR n (%)	CS n (%)
Age (in years), Mean (SD)	50.2 (22.3)	53.5 (18.2)	52.8 (10.6)	52.4 (18.6)	48.2 (19.2)	47.7 (18.9)	50.5 (18.9)	52.4 (21.5)
*p* value	*p* < 0.0001		*p* = 0.217		*p* = 0.40		*p* = 0.0016	
Gender								
Male	2038 (62.9)	2313 (59.4)	3695 (69.6)	1929 (68.7)	3503 (70.3)	930 (72.3)	1789 (71.4)	1568 (71.2)
Female	1202 (37.10)	1583 (40.6)	1610 (30.3)	875 (31.21)	1474 (29.6)	356 (27.7)	714 (28.5)	634 (28.7)
*p* value	*p* = 0.0024		*p* = 0.426		*p* = 0.18		*p* = 0.88	
Diabetes mellitus	914 (28.2)	1332 (34.1)	1670 (31.4)	941 (33.5)	1221 (24.5)	234 (18.2)	714 (28.4)	629 (28.5)
*p* value	*p* < 0.0001		*p* = 0.056		*p* < 0.0001		*p* = 0.91	
Presence of luminal device	711 (21.9)	372 (9.5)	1005 (18.9)	305 (10.8)	867 (17.43)	146 (11.3)	523 (20.8)	265 (12.0)
*p* value	*p* < 0.0001		*p* < 0.0001		*p* < 0.0001		*p* < 0.0001	
Chronic kidney diseases (CKD)	458 (14.1)	567 (14.5)	993 (18.7)	329 (11.7)	771 (15.4)	81 (6.2)	410 (16.4)	284 (12.9)
*p* value	*p* = 0.617		*p* < 0.0001		*p* < 0.0001		*p* = 0.003	
Active malignancy	497 (15.3)	789 (20.2)	689 (12.9)	713 (25.4)	304 (6.1)	196 (15.2)	240 (9.5)	556 (25.2)
*p* value	*p* < 0.0001		*p* < 0.0001		*p* < 0.0001		*p* < 0.0001	
Recent surgery (within 90 days)	590 (18.2)	415 (10.6)	668 (12.5)	193 (6.8)	482 (9.69)	96 (7.4)	368 (14.7)	183 (8.3)
*p* value	*p* < 0.0001		*p* < 0.0001		*p* = 0.006		*p* < 0.0001	

Data are presented as mean ± standard deviation (SD) for continuous variables and as n (%) for categorical variables. Continuous variable (Age) was compared using the independent-samples *t* test, while categorical variables (Gender and co-morbidities) were compared using the Pearson's Chi–Square Test of Independence. All tests were two-sided, and a *P* value <0.05 was considered statistically significant.

Mortality was higher across all the CR group pathogens compared to susceptible group and it was statistically significant. Among *E. coli* infections mortality was higher in the CR group (24.4% vs. 17.3%; *p* < 0.0001) corresponding to a relative risk of death of 1.41 (95% CI 1.13–1.70, *p* < 0.0001) and PAF for mortality was 15.8% for CR *E. coli* (95% CI 14.6–17.1). For *K. pneumoniae,* mortality was higher in CRKP infections compared with CSKP (31.2% vs 23.5%; *p* < 0.0001), corresponding to a relative risk of death of 1.33 (95% CI 1.18–1.51, *p* < 0.0001) and PAF for mortality was 17.5% (95% CI 16.5–18.6). Among *A. baumannii* infections mortality was higher in the CRAB group (37.9% vs. 32.8%; *p* = 0.0005), corresponding to a relative risk of death of 1.16 (95% CI 1.02–1.34, *p* < 0.0006) and PAF for mortality was 11.1% (95% CI 10.3–12.0). Among *P. aeruginosa*, mortality was higher in CRPA infections (28.9% vs. 20.2%; *p* < 0.0001), with a relative risk of death of 1.43 (95% CI 1.15–1.92, *p* < 0.0001) and PAF for mortality was 18.7% (95% CI 17.3–20.4) (Table 3).

**Table 3:** Pathogen wise clinical outcomes for patients infected with Carbapenem-resistant gram-negative infections.

	*E. coli*	*K. pneumoniae*	*A. baumannii*	*P. aeruginosa*
Length of hospital stay in days mean (SD)				
CR	23.1 (20.2)	25.7 (21.6)	25.2 (22.1)	21.0 (26.4)
CS	17.8 (17.5)	24.1 (21.7)	30.9 (26.8)	25.6 (25.7)
Difference (95% CI), *p* value	5.2 (4.40–6.18) *p* < 0.0001	1.6 (0.65–2.63) *P* = 0.002	−5.7 (−7.5 to −3.9) *p* < 0.0001	−4.6 (−6.4 to −2.8) *p* < 0.0001
Death, n (%)				
CR	790 (24.4)	1653 (31.2)	1891 (37.9)	723 (28.9)
CS	672 (17.3)	659 (23.5)	422 (32.8)	444 (20.2)
Difference (95% CI), *p* value	7.2 (5.16%–9.32%) *p* < 0.0001	7.6 (5.65–9.67) *p* < 0.0001	5.1 (2.2–7.9) *p* = 0.0005	8.7 (6.3–11.2%) *p* < 0.0001
Relative risk of mortality, 95% CI	1.41 (1.13–1.70)	1.33 (1.18–1.51)	1.16 (1.02–1.34)	1.43 (1.15–1.92)
Population Attributable Fraction (PAF) for mortality	15.8%	17.5%	11.1%	18.7%
Cost of therapy per patient in USD				
CR	420	587	655	702
CS	211	505	436	510

Data are presented as mean ± standard deviation (SD) for continuous variables and as n (%) for categorical variables. Continuous variables (length of stay) were compared using the independent-samples *t* test, while categorical variables (mortality) were compared using Pearson's chi-square test.

Relative risks (RRs), 95% confidence intervals (CIs), and *P* values for mortality were estimated using modified Poisson regression with adjustment for clustering by study site. All tests were two-sided, and a *P* value <0.05 was considered statistically significant.

PAF was calculated using Levin's formula based on the proportion of resistant infections and the unadjusted relative risk. The 95% confidence intervals were estimated using the delta method.

Patients with CR *E. coli* infections had a longer hospital stay than those with CS infections [23.1 (SD 20.2) vs 17.8 (SD 17.5); P < 0.0001] and incurred higher antibiotic treatment costs ($420 vs $211). For *K. pneumoniae*, the length of hospital stay was comparable between the CR and CS groups [25.7 (SD 21.6) vs. 24.1 (21.7); *p* = 0.002], although treatment costs were higher in the CR group ($587 vs $505). Patients with CRAB and CRPA infections had shorter hospital stays (25.2 vs. 30.9; *p* < 0.0001 and 21.0 vs 25.7; *p* < 0.0001 respectively) but substantially higher treatment costs than those with CS infections ($655 vs. $436 and $702 vs. $510 respectively). The shorter hospital stay observed among patients with CRAB and CRPA infections is likely attributable to the higher mortality in these groups (Table 3). Compared with the analysis including all specimen types, bloodstream infections were associated with substantially higher mortality, longer hospital stay, and greater treatment costs across all pathogens (Table 4).

**Table 4:** Pathogen wise clinical outcomes for patients infected with bloodstream associated gram-negative infections.

	*E. coli*	*K. pneumoniae*	*A. baumannii*	*P. aeruginosa*
Length of hospital stay in days mean (SD)				
CR	21.8 (18.2)	25.4 (20.8)	25.7 (26.4)	31.1 (25.8)
CS	15.8 (14.5)	25.5 (23.6)	32.3 (32.6)	24.6 (23.8)
Difference (95% CI), *p* value	6.0 (3.7–8.3) *p* < 0.0001	0.1 (−2.3 to 2.1) *p* = 0.93	−6.6 (−10.5 to −2.7) *p* = 0.001	6.5 (3.0–10.0) *p* = 0.001
Death, n (%)				
CR	245 (39.3)	553 (44.8)	562 (50.8)	158 (46.4)
CS	240 (23.8)	220 (36.0)	140 (42.6)	114 (32.8)
Difference (95% CI), *p* value	1.65 (1.41–1.92) *p* < 0.0001	8.8 (4.1–13.5) *p* = 0.0002	8.1 (2.3–13.9), *p* = 0.009	13.6 (6.4–20.9), *p* = 0.0002
Relative risk of mortality, 95% CI	1.65 (1.36–2.01)	1.24 (1.01–1.53)	1.19 (1.02–1.58)	1.41 (1.13–1.85)
Population Attributable Fraction (PAF) for mortality	19.8%	14.1%	12.8%	17.0%
Cost of therapy per patient in USD				
CR	724	781	582	739
CS	311	630	527	598

Data are presented as mean ± standard deviation (SD) for continuous variables and as n (%) for categorical variables. Continuous variables (length of stay) were compared using the independent-samples *t* test, while categorical variables (mortality) were compared using Pearson's chi-square test.

Relative risks (RRs), 95% confidence intervals (CIs), and *P* values for mortality were estimated using modified Poisson regression with adjustment for clustering by study site. All tests were two-sided, and a *P* value <0.05 was considered statistically significant.

PAF was calculated using Levin's formula based on the proportion of resistant infections and the unadjusted relative risk. The 95% confidence intervals were estimated using the delta method.

With respect to antimicrobial therapy, ceftazidime–avibactam–based regimens were used more frequently than polymyxin-based regimens for treating carbapenem resistant *E. coli* and *K. pneumoniae.* In contrast both the therapies were used equally for CR *P. aeruginosa*. Details of antimicrobial therapy and associated outcomes are summarised in Table 5. Treatment with Ceftazidime–avibactam was associated with lower mortality in patients having CR *E. coli* and *K. pneumoniae* infections. However, mortality was higher among patients with *P. aeruginosa* receiving ceftazidime–avibactam.

**Table 5:** Therapy administered and clinical outcome in patients with carbapenem resistant organisms.

	Polymyxin B	Ceftazidime-avibactam	Difference (95% CI)	p-value
All samples				
Carbapenem resistant *E. coli*				
n (%)	352 (12.2)	475 (16.5)	4.3 (2.5–6.4)	<0.0001
Duration of therapy in days mean (SD)	6.6 (6.1)	7.4 (6.5)	0.8 (0.4–1.1)	<0.0001
Mortality, n (%)	110 (31.2)	174 (36.6)	5.4 (−1.2-11.7)	0.106
Carbapenem resistant *Klebsiella pneumoniae*				
n (%)	1138 (19.5)	1543 (26.4)	6.9 (5.4–8.4)	<0.0001
Duration of therapy in days mean (SD)	7.1 (8.4)	7.7 (8.6)	0.6 (−0.5 to 1.2)	0.071
Mortality, n (%)	445 (39.1)	586 (37.9)	1.2 (−2.5 to 4.9)	0.527
Carbapenem resistant *Pseudomonas aeruginosa*				
n (%)	477 (24.8)	475 (24.7)	0.1 (−2.6 to 2.8)	0.942
Duration of therapy in days mean (SD)	7.2 (6.7)	9.0 (12.7)	1.8 (0.5–3.1)	0.006
Mortality, n (%)	167 (35.0)	202 (42.5)	7.5 (1.3–13.6)	0.017
Blood stream Infections				
Carbapenem resistant *E. coli*				
n (%)	99 (18.9)	168 (32)	13.1% (7.9–18.2)	<0.0001
Duration of therapy in days mean (SD)	6.4 (7.0)	6.0 (6.3)	−0.4 (−2.0 to 1.2)	0.631
Mortality, n (%)	36 (36.3)	61 (21.4)	14.9 (3.8–26.1)	0.008
Carbapenem resistant *Klebsiella pneumoniae*				
n (%)	267 (19.1)	544 (38.9)	19.8 (16.5–23.1)	<0.0001
Duration of therapy in days mean (SD)	6.2 (5.9)	7.0 (5.9)	0.8 (−0.1 to 1.6)	0.070
Mortality, n (%)	136 (50.9)	233 (42.8)	8.1 (0.8–15.3)	0.029
Carbapenem resistant *Pseudomonas aeruginosa*				
n (%)	66 (26.2)	82 (32.5)	6.3 (−1.6 to 14.1)	0.120
Duration of therapy in days mean (SD)	6.1 (7.4)	11.3 (25.5)	5.2 (−1.2 to 11.6)	0.111
Mortality, n (%)	25 (37.8)	46 (56.1)	18.3 (2.1–33.1)	0.027

Data are presented as n (%) for categorical variables and mean ± standard deviation (SD) for continuous variables. The proportion of patients receiving polymyxin B or ceftazidime-avibactam and mortality were compared using Pearson's chi-square test. Duration of therapy was compared using the independent-samples *t* test. Differences are presented as absolute percentage differences (for categorical variables) or mean differences (for continuous variables). All tests were two-sided, and a *P* value <0.05 was considered statistically significant.

## Discussion

In this multicentre hospital-based observational study across 20 tertiary care hospitals in India, we document that CR Gram-negative infections are associated with a higher risk of mortality, prolonged hospitalisation, and increased antibiotic treatment costs compared with phenotypically susceptible infections caused by the same pathogens. These findings were consistent across *E. coli*, *K. pneumoniae*, *A. baumannii*, and *P. aeruginosa*, with the greatest impact observed in bloodstream infections (39–51%) and infections due to non-fermenting Gram-negative organisms. Our findings provide evidence on hospital-based estimate for burden of AMR in India. Given that metallo-β-lactamases (MBLs) are the predominant mechanism of carbapenem resistance among Gram-negative pathogens in India,4^4^ these findings could potentially reflect the burden associated with MBL-producing organisms among hospitalised patients in India, however this study does not include such analysis.^12^

Estimating mortality attributable to carbapenem resistance is complex due to multiple confounding factors, including patient-level risk determinants, delays in initiating appropriate therapy, and limitations in the timeliness and capacity of existing diagnostic infrastructure to detect resistance. Consequently, the observed excess mortality in cohorts with CR infections may not accurately reflect the proportion of deaths that could be prevented in the absence of resistance. We calculated population attributable fraction (PAF) for mortality for each subgroup and report PAF of 15.8%, 17.5%, 11.1%, and 18.8% due to carbapenem resistance of *E. coli*, *K. pneumoniae*, *A. baumannii* and *P. aeruginosa* respectively. PAF estimates of CR *E.coli* in our study are similar to that reported by EURECA study.^13^ As per our estimates, deaths of 11%–19% of patients can be prevented if there was no carbapenem resistance. However, these estimates should be interpreted with caution, as PAF was calculated using the unadjusted relative risks and did not account for important patient-level confounders, including comorbidity status, patient severity indicators, time of appropriate therapy and source reduction/removal. More robustly designed studies incorporating detailed clinical severity indicators and adjusted effect estimates are needed to derive more accurate attributable burden estimates.

The cost of antibiotic therapy for CR Gram-negative bacteria infections was 1.1–2 fold higher compared with susceptible infections.^14^^,^^15^ Although our analysis included only direct antibiotic costs, these findings underscore the financial impact of antimicrobial resistance, likely attributable to prolonged treatment duration and the use of newer reserve antimicrobial agents. This increased cost inflicts a considerable burden on both patients and the healthcare system, particularly in settings where out-of-pocket healthcare expenditure is high.

We further evaluated the clinical outcomes among patients with CR organisms as per the antimicrobial therapy administered. Ceftazidime–avibactam–based therapy was associated with improved outcomes in CR *E. coli* and *K. pneumoniae* blood stream infections. Markedly higher mortality among patients infected with CRAB and CRPA (46.4–50.8%) was observed when compared with CR *E.coli* (39.3–44.8%). This observation is consistent with the growing evidence from other cohort studies which documented improved clinical response and lower mortality with ceftazidime–avibactam alone or in combination with aztreonam compared to polymyxin-based regimens.^16^, ^17^, ^18^ However, no clear benefit of ceftazidime–avibactam–based therapy was observed in CR *P. aeruginosa*, where the outcomes were similar or worse compared with polymyxin-based regimens. This finding resonates with the mixed evidence from the published studies, with some reporting superiority of ceftazidime–avibactam and others demonstrating comparable clinical efficacy, likely influenced by variability in the resistance mechanisms and clinical severity.^19^^,^^20^ The observed differences in therapeutic outcomes should also be interpreted in the context of underlying resistance mechanisms. Molecular characterisation studies undertaken as part of the broader ICMR-AMR surveillance network demonstrate heterogeneous carbapenemase profiles across pathogens in these hospitals. Among the CR *E. coli* and *K. pneumoniae*, NDM (14.0% and 14.9%, respectively) and OXA-48-like enzymes (11.9% and 25.6%, respectively) were commonly identified, whereas CR *P. aeruginosa* frequently harboured NDM (40%) and co-producer mechanisms (approximately 20%; e.g, NDM+VEB or NDM+GES). In CRAB, OXA-23 was predominant (43%), with frequent co-production involving OXA-23 plus NDM or TEM (44%).^21^ However, these molecular findings were derived from a subset of randomly selected isolates and were not linked to patient-level therapeutic outcomes in the present study.

The clinical outcomes across antimicrobial regimen administered in our study must be interpreted with caution, as several patient-related factors, including severity of illness, time to appropriate therapy, source control, and site of infection, as well as microbiological factors such as carbapenemase type and ceftazidime–avibactam–aztreonam synergy testing, were not evaluated at the patient level in the present analysis. Therefore, these findings are more reflective of the clinical practice in India rather than definitive evidence for the superiority of one antimicrobial regimen over another.

The length of hospital stay in patients with CR *E. coli* was higher compared to CS *E. coli* but it was shorter for CR *A. baumannii* and CR *P. aeruginosa*. This paradox likely reflects the early mortality in CRAB and CRPA infections, leading to reduced length of hospital stay owing to poor survival.

Our findings have several important policy implications. The findings highlight the need to move beyond the isolate-based surveillance towards an integrated surveillance linking microbiological data with clinical outcomes. Strengthening access to timely and accurate diagnostics is equally important, as it is essential for guiding appropriate antibiotic use and reducing unnecessary exposure to broad-spectrum agents. Countries should prioritise the scale up of antimicrobial stewardship and IPC interventions within the national health systems. Finally, improving access to newer effective antimicrobials, is crucial to reduce mortality associated with drug resistant infections in India.

Our study has several limitations. First, while culture requests were clinician-driven and therefore presumed to reflect true infections, some cases may represent colonisation rather than active infection. To mitigate this, we have presented bloodstream infection results separately. Second, we did not provide complete list and details of the treatment given to these patients, as the treatment protocols may have differed across centres study periods. Third, the economic assessment only takes into account the cost of antibiotics and does not include room/bed charges or supportive care like cost of diagnostic investigations, doctors’ consultation fees, which can differ substantially between institutions thereby underestimating the true financial burden.^22^ For uniformity, we have calculated costs as per the national rates for antibiotics provided under Government funded JanAushadhi scheme, which are generally lower than open-market prices and may have further resulted in conservative estimates of treatment costs. Fourth, the comparison of outcomes between treatment groups and contribution of carbapenem resistance to mortality is subject to confounding, as adjustments were not made for key clinical variables, including severity of illness, timing of appropriate therapy, source of infection, and resistance mechanisms. Fifth, there may be over inflation of length of stay and cost of care in patients with carbapenem resistant infections, as most of these infections happen after the patient is hospitalised and has survived initial reason for admission. We did not adjust both parameters for this factor. Finally, we report here only the all-cause mortality and not the attributable mortality caused by these pathogens, which may influence the precise estimation of infection-related outcomes.

Most of the hospitals, included in the study have very high standards of infection control and are accredited by national and international quality control boards, but they are tertiary care hospitals, which predominantly manage referred and critically ill patients; therefore, the rates observed should not be interpreted as indicators of hospital performance. We suspect that hospitals with poor laboratory support to facilitate timely identification and treatment may have far worse patient outcomes compared to what is documented in this study. It is possible that results of clinical outcomes presented here may actually be an underestimate. To our knowledge, this is one of the largest patient-level studies from India to quantify the burden of CR Gram-negative infections across multiple pathogens and clinical syndromes, addressing a critical gap in the evidence base.

In conclusion, carbapenem resistant Gram-negative infections impose substantial burden characterised by high mortality prolonged hospitalisation and higher treatment costs. These findings underscore the urgent need for coordinated action by policymakers, hospital administrators, and clinicians to strengthen antimicrobial stewardship, infection prevention, and access to effective therapies while preventing their misuse, to address the escalating antimicrobial resistance crisis in India. Without sustained intervention, the rising burden of carbapenem resistance threatens to undermine recent gains in infectious disease control and poses a major challenge to the health care systems in India.

## Contributors

NB: study design, data curation, data interpretation, formal analysis, review and editing; SV: data curation, data interpretation, formal analysis, and writing of the original draft, review and editing; VRS: study design, methodology, review; CR: study design, methodology, review; PK: development of online data entry portal, data management; BY: data collection and data analysis; VC: study design, review; KW: conceptualisation, study design, investigation, methodology, project administration, resources, supervision and writing of the original draft, review and editing. KW, NB and SV accessed and verified all data in this study. All authors had access to all the data in the study and had final responsibility for the decision to submit for publication. The consortium members were responsible for prospective data collection from all patients admitted to the designated 150 surveillance beds in their respective hospitals and for entering the required patient information into the study database in accordance with the study protocol.

## Data sharing statement

This study data was generated through an online data management system developed and maintained by the Indian Council of Medical Research (ICMR). The data supporting the findings of this study are not publicly available but may be accessed upon reasonable request. The corresponding author can be contacted for information on data availability.

## Declaration of interests

CR reports honoraria from Pfizer, Biomerieux, Cipla and Wockhardt for activities outside the submitted work. VRS reports speaker fees, advisory boards, travel and research grants from Pfizer, GSK, Mylan, Cipla, Glenmark, Zydus, BD, Biomerieux and Wockhardt for activities outside the submitted work. The authors declare that they have no other potential conflict of interests regarding this work.

## Appendix A Supplementary data

Supplementary Tables

Collab Authors
