# Supplementary material for: Mortality and cost of therapy of carbapenem susceptible vs resistant Gram-negative infections among hospitalised patients in India: a multicentric surveillance (2022–2025)
Source: Lancet Reg Health Southeast Asia. 2026 Jul 30;53:100829. doi: 10.1016/j.lansea.2026.100829 (PMC13546927; doi:10.1016/j.lansea.2026.100829)
Supplement: Supplementary file 1 [file mmc1.docx]

**SUPPLEMENTARY MATERIAL**

**Table 1: Study sites as a part of ICMR-Antimicrobial Resistance Surveillance Network**

| **Zones** | **States/Union Territories** | **Participating hospitals** |
| --- | --- | --- |
| South | Karnataka, Tamil Nadu, Kerala, Telangana, Puducherry | 6 |
| North | Rajasthan, Uttar Pradesh Jammu and Kashmir, Delhi, Chandigarh | 6 |
| East and North-East | West Bengal, Assam and Manipur | 4 |
| West | Maharashtra | 3 |
| Central | Madhya Pradesh | 1 |

**Text S1 – Classification of infection versus colonization**

**Definitions**

Any sample from a non-sterile site (respiratory secretions including sputum, endotracheal aspirate, tracheal aspirate or bronchial lavage, urine sample, superficial swab or pus aspirated any other site) should fit into these broader definitions of the clinical syndromes as mentioned below.

| Syndrome | Definition |
| --- | --- |
| Pneumonia  HAP/VAP | Criteria for pneumonia   - Fever or documented temperature ≥99.4 °F,   OR   - Cough (with or without sputum)   PLUS any one of the following   - Shortness of breath - Pleuritic chest pain - Lung signs (rales, crepts, pleuritic rub) - Hypoxia (sp02 of < 92% on room air) - New opacity in Xray Chest PA view - HAP is an acute bacterial pneumonia in a patient hospitalized for ≥48 hours or developing pneumonia within 7 days following discharge from a hospital. - Patients may suffer from acute respiratory failure and require mechanical ventilation (ventilated‑HAP). VAP is defined as acute bacterial pneumonia in a patient who is already receiving mechanical ventilation for ≥48 hours. |
| cIAI | The clinical diagnoses and brief descriptions that define the population of patients with cIAIs include, but are not limited to, the following:   - Intra-abdominal abscess: one or more abscesses surrounding diseased or perforated viscera often characterized by nonspecific abdominal pain - Perforation of intestine: an acute perforation of the intestine associated with diffuse infection of the peritoneum, often characterized by nonspecific abdominal pain - Peritonitis: a diffuse infection of the peritoneum, often characterized by nonspecific abdominal pain - Appendicitis with perforation or periappendiceal abscess: an acute infection of the appendix characterized by colicky abdominal pain often localized to the right lower quadrant - Cholecystitis with perforation or abscess: an acute infection extending beyond the gallbladder wall, often accompanied by right upper quadrant abdominal pain |
| cIAI | The clinical diagnoses and brief descriptions that define the population of patients with cIAIs include, but are not limited to, the following:   - Intra-abdominal abscess: one or more abscesses surrounding diseased or perforated viscera often characterized by nonspecific abdominal pain - Perforation of intestine: an acute perforation of the intestine associated with diffuse infection of the peritoneum, often characterized by nonspecific abdominal pain - Peritonitis: a diffuse infection of the peritoneum, often characterized by nonspecific abdominal pain - Appendicitis with perforation or periappendiceal abscess: an acute infection of the appendix characterized by colicky abdominal pain often localized to the right lower quadrant - Cholecystitis with perforation or abscess: an acute infection extending beyond the gallbladder wall, often accompanied by right upper quadrant abdominal pain |
| UTI | Cystitis  Presence of one or more of the following symptoms  1. Dysuria  2. Urinary frequency  3. Urinary urgency  4. Supra-pubic pain  There are no signs or symptoms that suggest an upper tract or systemic infection (as mentioned below)  Uncomplicated Pyelonephritis  Presence of anyone of the following:  1. Fever  2. Chills/rigors  3. Flank Pain  4. Costo-vertebral tenderness  AND    Any one symptoms suggestive of UTI (dysuria/frequency/urgency of urination) with no urinary tract abnormalities  Complicated UTI   - Men: Any form of UTI is complicated UTI - Women: Fits into clinical definition of Cystitis or uncomplicated pyelonephritis but presence of urological abnormalities or presence of Diabetes Mellitus or Pregnancy or renal transplant recipient or immunocompromising condition or presence of foreign body (DJ stent) in Urinary Tract. |
| SSSI (skin soft tissue infection) | An infection of the skin with a lesion size area of at least 75cm^2^ (lesion size measured by the area of redness, edema, or induration). A sufficiently large lesion size also differentiates between minor cutaneous abscess (smaller than approximately 75 cm^2^) and major cutaneous abscess (greater than approximately 75 cm^2^).  The following infection types are defined as SSSI:   - Cellulitis/erysipelas: A diffuse skin infection characterized by spreading areas of redness, edema, and/or induration - Wound infection: An infection characterized by purulent drainage from a wound with surrounding redness, edema, and/or induration - Major cutaneous abscess: An infection characterized by a collection of pus within the dermis or deeper that is accompanied by redness, edema, and/or induration |

BSI = bloodstream infection; cIAI= Complicated intra-abdominal infections; cUTI = complicated urinary tract infection; FDA = Food and Drug Administration; HAP = hospital-acquired pneumonia; IV = intravenous; LCBI = laboratory-confirmed bloodstream infection; SIRS = systemic inflammatory response syndrome; UTI = urinary tract infection; VAP = ventilator-associated pneumonia.

**Table S2-**Standard rate card for the antibiotics based on Government funded JanAushadhi

| Antibiotics | Rates (in Rs) | Designated rates as per DDD |
| --- | --- | --- |
| Meropenem (1gm) | 260 | 780 |
| Imipenem (500mg) | 300 | 1200 |
| Doripenem (500mg) | 500 | 1500 |
| Vancomycin (500mg) | 135 | 540 |
| Teicoplanin (400mg ) | 700 | 700 |
| Colistin (4.5MU) | 450 | 900 |
| Ceftazidime-avibactam (2.5g) | 4554 | 27324 |
| Fosfomycin (IV) 4gm | 1000 | 8000 |
| Polymyxin B 5 lac IV | 450 | 1350 |
| IV Linezolid | 110 | 220 |
| Tigecycline 50mg | 620 | 1240 |
| Minocyline (IV) 100mg | 1300 | 2600 |

**Text S2 –Formula for calculation of the 95% confidence interval for PAF**

**Text S3 -** Ethics approval numbers for all 20 hospitals

| **Hospitals** | **Ethical approval number** | **Ethical Committee name** |
| --- | --- | --- |
| H1 | IEC1/110/2022 | Institutional Ethics Committee |
| H2 | AMH-C-S-002/01-22 | Institutional Ethics Committee -Clinical studies |
| H3 | AMC/EC/1483 | Institutional Ethics Committee |
| H4 | No.165/Ethics/2022 | Institutional Ethics Committee |
| H5 | IHEC-LOP/2022/EL008 | Institutional Human Ethics Committee |
| H6 | IEC/2018/532 | Institutional Ethics Committee |
| H7 | IEC/2022/3894 | Institutional Ethics Committee |
| H8 | No.2022/CENT-GOVT/41/IRB18 | Institutional Review Board |
| H9 | No.IEC/1518/AL/22/14 | Institutional Ethics Committee |
| H10 | IEC-01/2022-2305 | Institutional Ethics Committee |
| H11 | IEC/2022/185 | Institutional Ethics Committee |
| H12 | No.IEC-320/01.04.2022 | Institutional Ethics Committee |
| H13 | No.EC/03/22/206 | Institutional Ethics Committee |
| H14 | JIP/IEC/2022/0147 | Institutional Ethics Committee |
| H15 | REB/Prop(FP)/181/109/21/2022 | Research Ethics Board |
| H16 | No.ECASM-AIMS-2022-046 | Institutional Ethics Committee |
| H17 | IRB/No./14752 | Institutional Review Board |
| H18 | No.IEC/2022/187 | Institutional Ethics Committee |
| H19 | EC/NIMS/3044/2022 | Institutional Ethics Committee |
| H20 | IEC/MED/10/2022 | Institutional Ethics Committee for Research on Human subjects |
