# Supplementary material for: Mortality and cost of therapy of carbapenem susceptible vs resistant Gram-negative infections among hospitalised patients in India: a multicentric surveillance (2022–2025)
Source: Lancet Reg Health Southeast Asia. 2026 Jul 30;53:100829. doi: 10.1016/j.lansea.2026.100829 (PMC13546927; doi:10.1016/j.lansea.2026.100829)
Supplement: Supplementary file 2 [file mmc2.docx]

**ICMR AMR Network collaborators**

| **First and middle M** | **Last name** |
| --- | --- |
| Ambuj | Yadav |
| Amit | Gupta |
| Amit | Kumar |
| Anju Susan | Jacob |
| Aparna | Chakraborty |
| Aparna | Sonowal |
| Apurba Sankar | Sastry |
| Arya | Kumar |
| Ashish | Bhalla |
| Ashok Kumar | Pannu |
| Ashwani | Gupta |
| Asim | Kundu |
| Ayesha | Sunavala |
| Babita | Gupta |
| Balaji | Veeraraghavan |
| Bashir | Fomda |
| Bhanu | Kumar |
| Bharti | Taksande |
| Binila | Chacko |
| Brijendra Kumar | Rao |
| Chand | Wattal |
| Chhavi | Sawhney |
| Chiranjay | Mukhopadhyay |
| D Himanshu | Reddy |
| Daisy | Phukan |
| Deepak | Aggarwal |
| Deepak | Gupta |
| Deepak | Kumar |
| Deepthi | Avvaru |
| Durga Shankar | Meena |
| Gopal Krishana | Bohra |
| Hari Prasad | Sirigiri |
| Hitendrapal | Solanki |
| Irom | Loha |
| Ishrat | Rashid |
| Jasper | Rathinam |
| Jaswinder Kaur | Oberoi |
| Kamal Uddin | Zaidi |
| Kamran | Farooque |
| Kapil Dev | Soni |
| Kavitha | Jayaram |
| Kiran | Maribashetti |
| Mamta | Kshetrimayum |
| Manisha | Biswal |
| Meera | John |
| Mitraa | Shyam |
| Mousumi |  |
| Muralidhar | Varma |
| N Sanjib | Singh |
| Narayanan | Parameswaran |
| Nasima | Ahmed |
| Navneet | Sharma |
| Neelam | Taneja |
| Nishad | Plakkal |
| Nusrat | Shafiq |
| Padmaja | Durga |
| Pankaj | Arora |
| Parijat | Das |
| Pradeep | Saxena |
| Pralay | Ghosh |
| Pranjal Kumar | Dutta |
| Prashant | Gupta |
| Purva | Mathur |
| Raja | Ray |
| Reema | Nath |
| RehanUl | Haq |
| Richa | Aggarwal |
| Ritin | Mohindra |
| Roohi | Mohiuddin |
| Rupamdeep | Kaur |
| Sadia | Khan |
| Sadik | Mohammed |
| Sagar | Khadanga |
| Sai | Shree |
| Saidulu | Ganta |
| Samir | Yelwatkar |
| Sanjeev | Singh |
| Sayli | Gore |
| Sheetal | Varma |
| Subhalakshmi | Das |
| Subodh | Kumar |
| Sudipta | Mukherjee |
| Sukanya | Vimala |
| Sushma | Sagar |
| Syed Mudasir | Qadri |
| Tadepalli | Karuna |
| T Jeeten Kumar | Singh |
| Vandana Kalwaje | Eshwara |
| Vanita | Jain |
| Venkateswaran | Ramanathan |
| Vibhor | Tak |
| Vijay | Sharma |
| Vineeta | Dhyani |
| Vijayshree | Deotale |
| Vivek | Handa |
| Vivek | Trikha |
| Wazid | Ali |
| Yamunadevi V | Ramanathan |
| Yogiraj | Roy |
